# Comparative indicators for cancer network management in England: Availability, characteristics and presentation

**DOI:** 10.1186/1472-6963-8-45

**Published:** 2008-02-27

**Authors:** Mark McCarthy, Arturo Gonzalez-Izquierdo, Chris Sherlaw-Johnson, Artak Khachatryan, Michel P Coleman, Bernard Rachet

**Affiliations:** 1 UCL Department of Epidemiology and Public Health, University College London, 1-19 Torrington Place, London, WC1E 6BT, UK; 2 UCL Clinical Operational Research Unit, Department of Mathematics, University College London, Gower Street, London, WC1E 6BT, UK; 3 Cancer Research UK Cancer Survival Group, London School of Hygiene & Tropical Medicine, Keppel Street, London, WC1E 7HT, UK

## Abstract

**Background:**

In 2000, the national cancer plan for England created 34 cancer networks, new organisational structures to coordinate services across populations varying between a half and three million people. We investigated the availability of data sets reflecting measures of structure, process and outcome that could be used to support network management.

**Methods:**

We investigated the properties of national data sets relating to four common cancers – breast, colorectal, lung and prostate. We reviewed the availability and completeness of these data sets, identified leading items within each set and put them into tables of the 34 cancer networks. We also investigated methods of presentation.

**Results:**

The Acute Hospitals Portfolio and the Cancer Standards Peer Review recorded structural characteristics at hospital and cancer service level. Process measures included Hospital Episode Statistics, recording admissions, and Hospital Waiting-List data. Patient outcome measures included the National Survey of Patient Satisfaction for cancer, and cancer survival, drawn from cancer registration. Data were drawn together to provide an exemplar indicator set a single network, and methods of graphical presentation were considered.

**Conclusion:**

While not as yet used together in practice, comparative indicators are available within the National Health Service in England for use in performance assessment by cancer networks.

## Background

"Assessment of the quality of cancer treatment and care depends upon the availability of accurate and relevant information about the process and outcomes of care for patients [1]."

Cancer is a leading cause of death and disability across the world, and cancer services consume a significant proportion of health care resources. In England, the Calman-Hine report [2] in 1995 prioritised improvement in the quality of cancer services up to international levels, and the English Department of Health developed a 'National Service Framework' for cancer. This Cancer Plan [3] led to appointment of a national Director of Cancer Services and national Cancer Action Team, responsible for implementation, a Cancer Services Collaborative tasked with improving hospital-level organization of services, a new managerial tier of 34 cancer networks to coordinate services between hospitals, and development of local tumour-specific multi-disciplinary teams to provide enhanced specialist care.

Cancer networks in England were chosen to reflect existing geographical patterns of referral and joint care for cancer patients, for example for radiotherapy and specialised surgery or chemotherapy. They cover populations varying between a half and 3 million people, and roughly following local administrative boundaries. They each have a small administrative team of 5–15 staff, some of whom are centrally funded and thereby accountable to the Cancer Action Team. Network members are determined locally, drawing together managerial staff from NHS hospitals and clinical staff collaborating in tumour-specific multi-disciplinary teams.

To underpin the Government's commitment to improving the quality of cancer services and modernising cancer care, the Director of Cancer Services issued a Cancer Information Strategy [4] designed to monitor progress towards achieving specific targets (such as waiting times) and reducing the cancer death rate. The Strategy recommended that '...cancer networks should develop appropriate structures and processes to improve the availability and quality of information for cancer patients and carers'; and that 'monitoring of performance indicators which relate to the quality of cancer services delivery, including screening, should form part of the assessment of individual cancer services.' The Strategy concluded that 'Health service managers and commissioners working at local ..., regional or national level will need information on the structure, process and outcome of cancer care in the area for which they are responsible.' This required 'information about cancer services and aggregated information on activity and outcomes.'

As part of a study to measure quality in cancer services [5], we have identified a set of relevant indicators that could be derived from existing data sources, and that could be used as performance measures by cancer networks. We report on the data sources and the availability, characteristics and presentation of these data sets.

## Methods

Donabedian [6] recommended analysis of health services according to structure (the resources and facilities for services), process (the activity of providing services) and outcome (the effect on the patient or population). Mainz [7] has also used this typology in reviewing use of clinical indicators for quality in health care. We identified national data sets from public sources – NHS, government agencies, and Department (Ministry) of Health – that related to one or more of these three elements, and were granted access the data sets by their primary owners. We investigated availability of data that had supported the Audit Commission Report on Cancer Services in England and Wales [8], but decided not to use this as the survey covered less than a quarter of the country. We gained data collected on palliative care services by a national charity, but we also decided not to use this as only some services had contributed data and the denominator populations were unknown.

Black et al [9] describe development of the Directory of Clinical Databases (DoCDat), an archive describing over 150 clinical databases in the UK. Fields recorded include general aspects (when it was set up, who it includes, geographical area it covers), data set (individuals and items included, security and confidentiality), outputs (who can analyse the data, audit reports, publications), management (who runs the data base, funding), quality of the data (aspects of coverage and accuracy) and contact details. Two of our six data sets were already recorded in DoCDat (for their national use) – cancer registration and Hospital Episode Statistics. We drew reports for these two from DoCDat, and used the format for assessing the other four data sets.

We requested, where appropriate, only data for four cancers – breast, colo-rectal, lung, prostate. One data set – the English Healthcare Commission's Acute Hospital Portfolio [10] – does not identify cancer patients or services separately, but included data on hospital structures that are relevant. We assessed the completeness of the data, excluding variables where incompleteness was a significant problem, (usually more than 10%). For some data sets, data were missing either as individual items or for the hospital as a whole. We made numerical checks to confirm that each variable's data were within appropriate ranges. We looked at distributions to determine outliers and, where data were normally distributed, calculated 5% confidence limits.

## Results

Six data sets forming the 'Cancer Networks Limited Data Set' are described. The Tables present the indicators for each data set, but only for one cancer each (for brevity).

### Acute Hospital Portfolio

Information about health services in England were collected by the Audit Commission from 2000/01, and have been continued thereafter annually by the Healthcare Commission [10]. Each survey addresses a different aspect of healthcare provision, including financial, facilities, structural and personnel. In the period for our study, we identified four relevant sets: medical staffing, ward statistics, radiology and medicines management. The 1999/2000 and 2000/2001 surveys were made on 188 NHS acute hospital trusts. There were explicit rules for deciding how to record variables, and data sets were estimated as mostly more than 80% complete. We chose a small number of relevant indicators from each set (Tables 1, 2, 3, 4).

**Table 1:** Acute Hospital Portfolio indicators for hospitals in the network: Medical Staffing

	**Hospitals**						
	
**Indicators**	**A**	**B**	**C**	**D**	**E**	**F**	**G**
Consultant WTE* per 1000 all admissions	2.12	5.45	2.04	n/a	2.31	2.26	n/a
Anaesthetist Consultant WTE per 1000 surgical admissions	0.85	1.79	1.01	n/a	0.83	1.31	n/a
Medicine Consultant WTE per 1000 medicine admissions	2.11	1.79	1.60	n/a	1.37	2.34	n/a
Pathology Consultant WTE per 1000 all admissions	1.88	0.98	2.92	n/a	2.60	2.00	n/a
Radiology Consultant WTE per 1000 all admissions	2.04	0.34	2.46	n/a	2.49	2.17	n/a
Ratio of outpatients to all admissions	4.08	3.55	5.15	n/a	4.64	4.70	n/a

* Whole-time equivalent (WTE)

n/a = not available

**Table 2:** Acute Hospital Portfolio indicators for hospitals in the network: Ward Statistics 2000/2001

	**Hospitals**						
	
**Indicators**	**A**	**B**	**C**	**D**	**E**	**F**	**G**
Clinical nurse specialists (WTE) per 1000 FCE*	n/a	2.20	0.87	0.32	0.80	0.65	0.50
Standardised ward patient accidents per 100 available beds	n/a	202	59	26	115	66	119
All formal complaints per 1000 FCEs	n/a	9.92	12.86	9.16	9.98	8.16	4.86

* Finished consultant episode (FCE)

**Table 3:** Acute Hospital Portfolio indicators for hospitals in the network: Radiology 2001/2002.

	**Hospitals**						
	
**Indicators**	**A**	**B**	**C**	**D**	**E**	**F**	**G**
Waiting times* – mammography	5	n/a	2	0	1	n/a	2
Waiting times* – nuclear medicine	6	4	2	n/a	6	n/a	n/a
Waiting times* – computer tomography	5	4	8	3	5	n/a	4
Waiting times* – magnetic resonance imaging	25	28	78	26	16	n/a	10
% exams unreported**	27	n/a	5	32	5	n/a	33
% exams reported by radiology staff	73	21	90	28	75	n/a	28
Inpatient exams per FCE	0.44	n/a	0.48	1.15	0.52	n/a	1.00
Outpatient exams per outpatient visit	0.25	n/a	0.23	1.23	0.29	n/a	1.33
Radiographers per 1000 FCE	0.71	n/a	0.71	1.24	0.73	n/a	1.48

*Average wait (median) in weeks

**Percentage of examinations unreported or reported by referring clinicians without agreement with radiology department

n/a = not available

**Table 4:** Acute Hospital Portfolio indicators for hospitals in the network: Medicines Expenditure 2001/2002.

	**Hospitals**						
	
**Indicators**	**A**	**B**	**C**	**D**	**E**	**F**	**G**
BNF* Spend malignant disease (£000)	n/a*	472	1620	2507	469	297	1963
BNF* Spend malignant disease per FCE** (£)	n/a	21.59	40.38	28.60	10.67	8.46	32.44

*British National Formulary (BNF)

### Cancer Services Peer Review

Along with the NHS Cancer Plan for England, a Manual for Cancer Services Standards was developed by the Department of Health. Approximately 170 hospital cancer units and centres were asked to assess themselves against these standards, and were then visited by teams of health care professionals and managers with expertise in the day-to-day delivery of cancer care, and also patient representatives. The visits were organised by each of the 13 NHS regions, and data collected uniformly (except for one region, Trent, which had piloted a different instrument). The review teams assessed the presence or absence of over 180 variables grouped in 10 areas: patient centred care, multi-disciplinary teams (for breast, colo-rectal and lung cancers), diagnostic services, oncology, radiotherapy, chemotherapy, palliative care, training, communication, and organisation. Through the detailed recording methods, all variables were at least 95% complete. For the Cancer Networks Limited Data Set, standards were selected in two areas:

• Compliance with all cancer standards for each main theme area – 12 variables (Table 5).

**Table 5:** Cancer Standards Peer Review indicators for hospitals in the network: number of standards achieved by themes

**Themes**	**Total number of standards**	**Hospitals**				
		
		**A**	**B**	**C**	**D**	**E**
Patient-centred care	*5*	1	0	2	2	3
Breast cancer MDT*	*39*	26.5	25	22	19	27
Colorectal cancer MDT*	*35*	23	19	15	23	24
Lung cancer MDT*	*36*	14	25	24	18	22
Pathology	*7*	4	3	5	4	4
Non-surgical oncology	*5*	1.5	1	0	0	0
Radiotherapy	*60*	(no service)	45	52	(no service)	59
Chemotherapy	*45*	21.5	30	33	37	34
Palliative care	*11*	8	6	11	7	7
Education	*2*	0	0	0	0	0
Communication	*3*	1.5	0	2	0	2
Cancer units (centres)	*15 (16)*	7	10	0	12	0

* Multi-disciplinary team (MDT)

• Compliance with multi-disciplinary team (MDT) theme variables (cancer-specific), grouped according to the sub-themes specified within the Manual – for breast 11 variables, (Table 6), colorectal 10 variables, and lung 10 variables.

**Table 6:** Cancer Standards Peer Review indicators for hospitals in the network: breast cancer multi-disciplinary team service standards – number of standards complied with.

**Topic**	**Total number of standards**	**Hospitals**				
		
		**A**	**B**	**C**	**D**	**E**
MDT structure	*9*	9	7	5	6	7
MDT meetings	*4*	1.5	3	1	2	3
Operational policies	*13*	6.5	7	8	7	8
Patient centred care	*5*	3	1	0	1	2
Treatment	*1*	1	1	1	0	1
Clinical guidelines	*1*	1	1	1	1	1
Referal guidelines	*1*	1	1	1	1	1
Data collection	*2*	2	2	2	0	2
Network audit	*1*	0	0	1	0	0
Clinical trials	*1*	0.5	1	1	1	1
MDT workload	*1*	1	1	1	0	1

* Multi-disciplinary team (MDT

### Hospital Episode Statistics

These data are collected routinely in all NHS hospitals and units, and record more than 12 million in-patient episodes per year. Data are held electronically by the NHS Health and Social Care Information Centre, a not-for-profit agency run by the National Health Service. Within a large data set, in which episodes can be linked within-year (but not across years), data were chosen for patients with a cancer diagnosis and data on length of admission by hospital, operation and consultant specialty (Table 7). Definitions of variables held are in the HES Data Dictionary. A national study in 2002 indicated 99% coverage of admissions [9], but for the variable 'mean and median length of stay' in the Limited Data Set, missing data by diagnosis was: breast 9.3%, colo-rectal 5.7%, lung 13.3%, prostate 6.5%.

**Table 7:** Hospital Episode Statistics indicators for hospitals in the network: colorectal cancers

**Indicators**	**Hospitals**					
	
	**A**	**B**	**C**	**D**	**E**	**F**
Number of patients	207	247	152	209	107	210
In-hospital mortality	13.5%	7.3%	9.9%	11.5%	16.8%	7.6%
Mean number of episodes per patient	1.2	6.1	1.6	6.5	5.1	3.4
Mean length of stay (days)	15.5	8.5	21.2	8.2	11.3	10.2
Median length of stay (days)	10	0	11	1	8	2
Mean waiting time (days)*	40.2	15.0	20.2	24.3	9.3	15.4

### Cancer Waiting Times

These data are collected by all NHS acute hospital trusts in England. For each trust, the data recorded in 2001/2002 were the percentage of GP urgent referrals achieving a waiting time of less than or equal 2 weeks (by tumour type – one month for some rarer cancers). However, measuring patients referred with a possible cancer diagnosis both includes non-cancer patients (wrong initial diagnosis) and excludes those identified by other routes (about half of all cancers). There are clear definitions and coding rules, but local completeness is unassessed, and data are missing for some trusts. The data are reported quarterly [11]. Table 8 shows returns for three years for lower gastro-intestinal cancer (ICD-10 codes C17-21 and C26, including malignant neoplasms of small intestine, colon, rectum, anus, and other and ill-defined digestive organs).

**Table 8:** Cancer Waiting Times indicators for hospitals in the network: lower gastro-intestinal tumours. Percentage of urgent GP referrals seen within 2 weeks (numbers of referrals in brackets)

**Tumour**	**Hospitals**						
	
	**Year**	**A**	**B**	**C**	**D**	**E**	**F**
Lower gastrointestinal**	2001/2*	98.1% (*441*)	100% (*90*)	97.8% (*47*)	89.7% (*156*)	94.6% (*75*)	97.5% (*40*)
Lower gastrointestinal**	2002/3	89.9% (*525*)	98.1% (*110*)	100% (*122*)	87.3% (*197*)	88.0% (*201*)	97.3% (*114*)
Lower gastrointestinal**	2003/4	100% (*441*)	100% (*157*)	98.3% (*186*)	99.6% (*271*)	97.7% (*177*)	100% (*184*)

*data includet only urgent referrals received within 24 hours

**ICD-10 codes C17-21 and C26

### Cancer survival

The 9 regional cancer registries in England collect population-based data on incidence and mortality from cancer. A subset of the data collected by the regional cancer registries is collated centrally to provide national figures on cancer incidence and survival. Regional cancer registries receive notification of incident cancer cases from sources including hospital in-patient and out-patient systems; radiotherapy; pathology; GPs, coroners; and chest clinics. Linkage between cancer registration and death certificates is achieved through the NHS Central Registry, which notifies cancer registries of registered patients who die with any diagnosis, and all patients with a cancer diagnosis. Registries check hospital case-notes of patients, and are estimated to include above 90% of patients with date of diagnosis as well as date of death (both are needed to calculate survival). The data may be analysed at sub-national levels [12], but for the present study it was concluded that data at cancer network level, with populations over 0.5 million, would be necessary to ensure statistical confidence when using relative survival analysis. For a limited group of individual level variables, including tumour types and place of treatment, there is 95% completeness, but lower figures for cancer stage. Table 9 shows relative survival rates (ie adjusted for other 'background' mortality) for lung cancer in males and females.

**Table 9:** Cancer Registry on survival (available only at network level). Age standardised relative survival estimates for patients diagnosed in 1996–2001 and followed-up till 31 December 2003

**Lung cancer**	**Sex**	**Cancer cases**	**Survival period**	**Relative survival, %**	**95% Confidence interval**
	male	1749	one-year	25.9	23.8
	female	1096	one-year	30.0	27.1
	all	2845	one-year	27.4	25.7
	male	1749	five-year	7.0	5.6
	female	1096	five-year	7.4	5.5
	all	2845	five-year	7.1	5.9

### National Cancer Patient Survey

This was a one-off survey of a sample of patients discharged from NHS acute hospitals in England [13]. The survey was undertaken by postal survey retrospectively in 2001, with reports from 56436 patients discharged between July 1999 – June 2000. Sampling and surveying were undertaken by an independent organisation, and the data stored in Economic and Social Research Council archive. The survey covered six cancers – breast, colorectal, lung, non-Hodgkin lymphoma, and ovary prostate. Response rates by trust were 60%-80%, while variables have 5–19% missing data. The survey asked patients their perspectives on care across the 'patient pathway': access to care, waiting times, diagnostic process, first treatment, hospital environment, outpatient experience. Table 10 shows a selection of these items for the Limited Data Set.

**Table 10:** Indicators from the National Survey of Cancer Patients for hospitals in the network. Prostate cancer: % respondents expressing a problem with each aspect of care.

**Question**	**Hospitals**					
	
	**A**	**B**	**C**	**D**	**E**	**F**
*Number of patients responding to different questions (range)*	*41 – 58*	*2 – 13*	*22 – 40*	*22 – 89*	*9 – 20*	*20 – 44*
Enough nurses on duty (a3)	20.6%	0.0%	27.5%	22.4%	21.0%	25.0%
Date of first hospital visit ever cancelled or postponed (b1)	11.1%	7.6%	14.7%	11.2%	25.0%	26.9% *
Treated with respect and dignity (b7)	21.2%	46.1% *	36.3% *	20.3%	26.3%	25.0%
Purpose of treatment discussed (b19	17.0%	38.4%	29.4%	23.4%	26.3%	14.8%
Consent form signed (b21)	20.4%	15.3%	0.0% *	25.3%	10.0%	15.3%
Pain and discomfort (b24)	59.5%	76.9%	81.8% *	65.0%	85.0% *	71.4%
Enough time explaining what would happen after discharge (c1)	18.6%	23.0%	36.3%	26.9%	36.8%	29.6%
Written or printed information given (c2)	36.3%	53.8%	48.4%	38.0%	36.8%	22.2%
Waiting time from GP visit till first hospital appointment (d3)	50.0%	50.0%	40.9%	61.7%	50.0%	55.0%
Being told what was wrong (d10)	1.8%	0.0%	6.6%	2.3%	5.8%	0.0%
Time spent telling what was wrong (d15)	33.9%	66.6%	32.2%	29.5%	33.3%	26.0%
Outpatient appointment ever cancelled or postponed by hospital (e4)	26.8%	16.6%	10.0%	14.8%	30.0%	24.3%
Waiting after appointment time to see a doctor (e6)	43.9%	33.3%	51.7% *	43.4%	33.3%	39.0%
Time spent by doctor during appointment (e7)	26.1%	0.0%	27.5%	22.7%	22.2%	26.8%
Whether time spent by doctor was enough (e8)	4.7%	16.6%	20.6% *	0.0%	11.1%	7.3%
Confidence and trust in doctor (e9)	0.0%	0.0%	6.8%	0.0%	11.1%	7.3%
Privacy (e11)	0.0%	0.0%	6.8% *	0.0%	0.0%	0.0%
Treated with respect and dignity (e13)	0.0%	0.0%	3.4%	0.0%	0.0%	0.0%

* Trusts significantly different from national average (p < 0.05)

### Data set characteristics

Three data sets (cancer registration/survival, HES, satisfaction) are recorded for individual patients and three (waiting times, Peer Review, Audit Commission) have data aggregated at hospital trust level. (Individual patient survival and hospital episodes cannot be routinely linked by the unique identifiers yet in England.) We could divide most of the data sets according to the four most common tumour types, breast, colo-rectal, lung and prostate cancers, which relate to the different natural survival patterns and different management requirements described in the national Manual of Cancer Services. Socio-economic position can be estimated from three-digit postcodes for data sets with individual records.

The data sets varied in size. Hospital Episode Statistics record more than 12 million episodes each year and the Audit Commission collected over 100 items of data from more than 200 trusts over a year. In contrast the Waiting Times survey concentrates only on this dimension for the same trusts. Only a few items were relevant from the large data set within Hospital Episode Statistics, describing hospital activity. The Audit Commission hospital data provided an array of general characteristics: a subset was chosen which was more particularly related to cancer (although not separated by tumour type). The Peer Review data were treated as binary (compliant or not compliant with the standard), and were summed to give total scores. The National Cancer Satisfaction Survey items had been chosen by an earlier factor analysis into nine themes each represented by one lead question, while five independent questions for one theme were averaged [13].

### Presentation

Where available, cancer service indicators can be presented with a mean and confidence interval, customarily set at 95%, as a snap-shot (cross sectional) or time related (longitudinal). Both may be presented in the presence or absence of a meaningful and appropriate comparator.

#### Cross sectional

**Figure 1:**
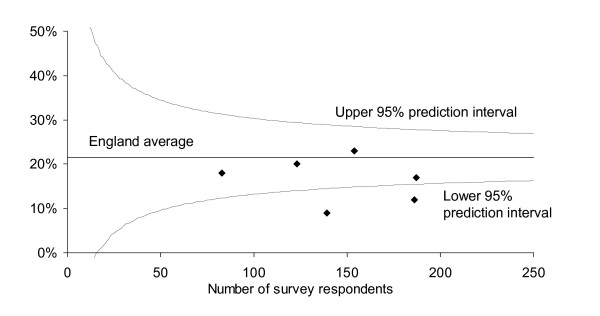
**Percentage of patients reporting 'a problem with amount of respect and dignity' in the National Cancer Patient Survey.** Values for trusts in one network

Figure 1 utilises the first approach and shows a funnel plot of an item from the patient satisfaction survey: each point is a trust. The plot takes into account the different sample sizes in each survey to indicate the normal confidence interval, and identifies outliers. It represents a snap-shot and contains data values that can be compared.

**Figure 2:**
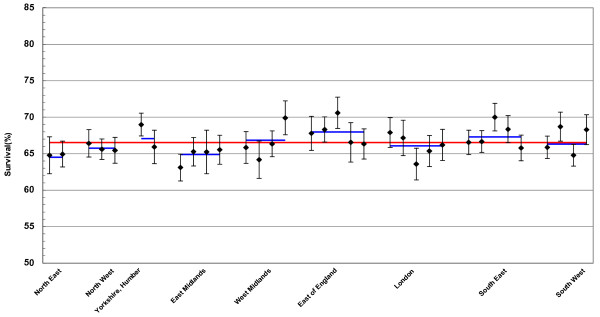
Breast cancer, age standardised five-year % relative survival (with CIs and national and regional averages) by cancer networks in England, grouped by NHS regions, of women with breast cancer diagnosed 1996–2001, followed up to 31 December 2003

Alternatively, points and confidence intervals can be arrayed individually against the group average, as shown in Figure 2 representing cancer survival at cancer network level, grouped by regions. This also represents a snap-shot and contains data values that can be compared.

**Figure 3:**
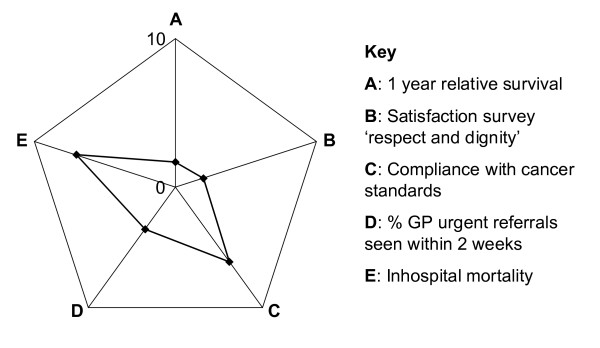
**Spider-chart ranking of one network for one dimension in each of five data sets for breast cancer (each dimension: 0 = most favourable, 10 = least favourable)**. **A**: 1-year relative survival. **B**: Satisfaction survey 'respect and dignity'. **C**: Compliance with cancer standards. **D**: % GP urgent referrals seen within 2 weeks. **E**: Inhospital mortality.

Lastly, the cancer indicators can be used together for comparisons: Figure 3 shows a spider plot of rankings for an item in each of the five data for a single network: best comparative performance would cover the least surface area, worst performance would cover the greatest area. This also represents a snap-shot but does not contain data values that can be compared – it is therefore a profile. Although this presentation does not show confidence intervals, the representation on a single plot of different dimensions allows visual assessment of relative closeness to stronger or weaker performance.

#### Longitudinal

**Figure 4:**
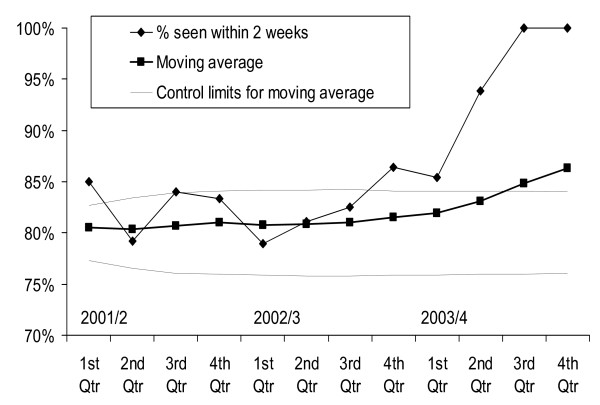
**Moving average control chart. Percentage GP urgent referrals for lung cancer seen within 2 weeks, single NHS hospital, data for consecutive year quarters 2001/2 – 2003/4**. % seen within 2 weeks. Moving average. Control limits for moving average

For monitoring, data can be used to review performance over time. Figure 4 shows a moving average control chart for cancer waiting times, with an expected trend and confidence intervals, based on the first year and a half, and with later observations moving strongly outside the projection.

## Discussion

Health service management decisions deserve to made with a full perspective of structural, process and outcome information. In the UK, cancer, uniquely as a disease group, has population incidence and outcome (survival) data available through cancer registers. Our study shows that other indicators can be drawn from data sets, describing hospital level characteristics, and the indicators can be presented together for management and comparative uses by cancer networks. While this study is theoretical, as these data are not at present being used together in this way in England, it demonstrates the potential for use in cancer service performance management at system level.

### Indicators

The NHS Cancer Plan [3] created a new organisational structure, the cancer networks, with a managerial objective of improving cancer care. While information for management was part of the original cancer strategy, we are not aware of any guidance provided to cancer networks for using information in quality improvement. Our study has identified cancer data sets that, with varying dimensions, can contribute to an assessment of service quality. Health care indicators have been developed in England to illustrate variations in practice and outcomes, for example the Healthcare Commission's performance ratings [14]. We identified six datasets from routine sources in England that reflect contemporary recommendations for broad-based indicators of quality [15,16]. Of the six data sets we identified, three (Hospital Episode Statistics, Waiting Times and cancer survival) are continuously collected, and collection of the Peer Review and Acute Hospitals Portfolio datasets is being repeated. The National Cancer Satisfaction Survey was only undertaken in full once, in 2000/2001, but there are now annual surveys of hospital patients, by Trusts in England, undertaken by the Healthcare Commission from which data on cancer patients could be drawn.

As NHS datasets are primarily collected for national rather than local use, there must be caution in using them in disaggregated form. For example, Rachet et al [12] consider that one and five year survival divided by tumour type and sex should not be presented as an indicator at the level of primary care organisation because of small numbers; however, at SHA level, and cancer network, these data can be presented with confidence intervals. Equally, the National Cancer Services Analysis Team [17] suggest that publication of Hospital Episode Statistics should be limited to Health Authority (and thus cancer network) level, on grounds of patient confidentiality, while more detailed analysis should be kept within the health service.

There is some overlap between the data sets: for example, cancer waiting times that are collected specially by Trusts refer to patients urgently referred for assessment by GPs, while hospital admissions data from Hospital Episode Statistics include all cancer patients admitted from a waiting list. These will, therefore, have slightly different meaning for management purposes. There are also different denominator populations: trusts serve catchment populations rather than geographical populations, and cancer networks are designed to reflect cooperation patterns between hospitals rather than strict administrative boundaries.

The use of cancer services indicators differs between stakeholders. The first use should be for staff who are providing the services – to understand it in aggregate, to see trends and to compare with others. This can lead to collective management decisions to address weaknesses suggested by the indicators, which may include further investigation and changes practice or provision. Within a managed system which is publicly accountable, such as the NHS, indicators can also be of use in monitoring – both identifying trends and assessing performance. The public are also potential users of such information, for example, in making choices between providers, although patients may be interested in information at a level of detail in relation to their own condition that is not available from routine aggregate indicators. A final set of stakeholders is the research community, since associations of data items between data sets may be used for explanatory studies of organisational determinants of care outcomes.

### Presentation

Indicators can enable public health practitioners, working alongside clinicians and managers, to assess the effectiveness and efficiency of services in improving population health. Flowers et al [18] have proposed '20 questions to ask a proposed public health indicator'. Bird et al [19] describe issues to increase the rigour of performance monitoring, and to limit inappropriate inferences. Equally, Spiegelhalter [20] has been concerned to ensure interpretation that fully recognises statistical variation.

The graphs present comparative data for cancer networks. The Department of Health has presented 'performance indicators' grouped the large number of hospitals into clusters (by size, teaching status, specialty etc) so as better to compare 'like with like'. It is also possible to set a finite level as an optimum 'target' for comparisons. However, there were fewer cancer networks than hospitals for us to compare, and we had no particular criteria to group as similarities or expected levels to set as targets.

Graphical methods of presentation help interpretation of statistical variation. Funnel plots are particularly relevant for presenting the satisfaction data, as they indicate critical differences in sample size. Caterpillar plots are more sophisticated than standard league tables, providing relevant comparisons, eg grouping by socio-economic characteristics or standardised by local populations. The control chart is a valuable method for assessing local trends in relation to specific management objectives. Spider-web diagrams allow easy comparisons across multiple dimensions, which is relevant for performance measurement.

### Quality monitoring

Performance indicators have developed because of greater availability of quantitative data, and can contribute to improving health care quality [16,18]. However, Freeman & Walshe [21] and Lilford et al [15] contrast use for external performance review with internal use for quality improvement. Hierarchical managers, for example government ministries, are interested in how health-care providers compare with each other and whether they are collectively achieving goals. Service providers are more interested in assessing how well they are performing and moving towards their own management goals.

There is broad agreement that measures of quality should be based on a collection of indicators rather than a single item or 'league table': a collection of indicators (sometimes called a 'scorecard') can better describe quality and provide different dimensions for each service to prioritise. Focussing on a limited number of targets may also encourage gaming.

Lilford et al [15] describe a range of studies linking quality of clinical care with outcomes, across various specialties and services, and conclude that the relationship is often weak or indeed non-existent. They propose that 'comparative outcome data should not be used by external agents to make judgments about the quality of hospital care'. Instead, they recommend that clinical services should 'monitor their own performance (process and outcome), compare themselves with others or their own past performance as appropriate, and take whatever action seems necessary'. For example, in a study in another clinical field, acute myocardial infarction, heart failure, and pneumonia hospital performance was improved through quarterly monitoring and feedback of standardized measures [22]. However, Lilford et al [15] also conclude that the literature shows stronger associations between clinical outcomes and organizational factors, including availability of equipment, staffing levels, management processes and staff communication. As decisions on resources may be made externally, for example by a higher-level tier, information from the data sets will need to be interpreted jointly between internal and external managers.

An intermediate use for indicators, between external monitoring and internal quality improvement, is in describing needs and trajectories for managers who are making investment decisions and who are accountable for population outcomes. The primary focus for information strategy in the NHS is the National Programme for IT ('Connecting for Health'), which seeks to link over 30,000 GPs in England with almost 300 hospitals, and give patients access to their personal health and care information. Nevertheless, this is a complex task, and the changing technology and fragmentation of providers (across such a large organisation as the NHS) provide many challenges to implementation.

### Improving cancer information

Cancer networks are a new approach to improving cancer services, based on the concept of coordination and development rather than hierarchical 'command' management. Their practice conforms with the findings of Leggat [23] that 'improving practice within hospitals ... the three most important aspects being the development of teamwork, performance management and sophisticated training'. Evaluations [24,25] suggest that cancer networks are developing successfully, but there are variations in the perceptions of need for, and use of, information for their work.

The National Health Service has a mixed track record in developing information systems [26]. It is a considerable challenge, because of the myriad of fields from which data can be recorded, and the variety of end-users. Moreover, the requirements of confidentiality, changing technology and market-led implementation have constrained free data exchange. But existing data sets about cancer services can be of use for local providers, monitoring and commissioning authorities, patients and researchers. Availability to users can be facilitated through the NHS National Programme for IT 'Connecting for Health'. However, this will require positive action by the Department of Health, as at present there are few incentives for networks to use or disseminate comparative aggregate data.

We might also envisage a geographically wider use of cancer data sets. In the USA, the National Cancer Institute has recognised the potential of 'cancer performance measures' to 'inform health policy and monitor cancer disparities and disease burden' at the macro-level [27].

In Europe, the EUROCARE study [28], showing the relatively poorer survival of cancer patients in Britain compared with European neighbours, was a trigger for the reorganisation of services in England and Wales in 1995. Another study, the European cancer health indicators project [29] has, within five fields of indicators, one on 'treatment & clinical aspects', suggesting that international indicators should include delay in cancer treatment (pilot studies), provision of radiotherapy and CT equipment, compliance with best oncology practice and percentage patients receiving palliative radiotherapy. While international comparisons must give attention to country differences in definitions and standards, indicators may also assist in understanding the relationship between the organisation of cancer services and differences in survival across Europe.

## Conclusion

The study has identified six contrasting datasets available to the National Health Service in England from which indicators can be drawn describing structure, process and outcomes for cancer services. At present, these indicators are not used together dynamically for comparisons or management by cancer networks, but the study demonstrates their potential for integrated use at system level.

## Competing interests

The author(s) declare that they have no competing interests.

## Authors' contributions

MM conceived the study, oversaw its implementation and wrote the manuscript. AG-I prepared and managed the data sets with CS-J, who also made the example presentations. AK reviewed the dataset properties using DoCDat. MC and BR made the cancer survival analyses. All authors were members for the Measures of Quality in Cancer Services study, and have read and approved the final version.

## Pre-publication history

The pre-publication history for this paper can be accessed here:


